# Conceptual competence in medicine: promoting psychosomatic awareness in clinics, research and education

**DOI:** 10.3389/fpsyt.2025.1500638

**Published:** 2025-02-06

**Authors:** Dirk von Boetticher

**Affiliations:** Department of Psychosomatic Medicine and Psychotherapy, University Medical Centre Göttingen, Gööttingen, Germany

**Keywords:** conceptual competence, conceptual research, mind-body relation, biopsychosocial model, psychosomatic medicine

## Abstract

**Introduction:**

In recent decades, psychosomatic medicine has developed into a distinct specialty, bringing specific clinical concepts to bear seeking to acknowledge the unity (not the identity) of the mind and body in clinical care. Such concepts form the identity of the psychosomatic field as a distinct discipline and its epistemological status between somatic medicine and psychiatry. Despite the importance of these concepts from an educational and a research perspective, too little attention has been paid to their clinical impact.

**Methods:**

This paper investigated the general nature of concepts and their role and significance in structuring the clinical encounter and care, including consideration of their relevance for the hidden curriculum.

**Results:**

Conceptual competence is defined as a transformative awareness of the multilayered, fallible, and plural nature of human concepts, which have both descriptive and evaluative and action-guiding properties having both an explicit and an implicit meaning. Conceptual competence in psychosomatic medicine entails dealing competently with the mind–body–distinction and the biopsychosocial model (and criticism of it) with respect to the clinical situation.

**Discussion:**

Conceptual research is presented as an autonomous research area and the complement of empirical research, having a descriptive and a normative function: descriptively analyzing the concepts we have and normatively searching for the concepts that we need for the integrated care we strive for.

## 1 Introduction

The physician’s encounter with patients and their suffering is largely shaped by the clinical model and its concepts, which organize the doctor’s attitude, knowledge, and experience (1). Beginning with the birth of psychosomatics in the 1930s, its clinical, theoretical, and institutional evolution has taken place along with the development of certain necessary concepts. The identity of psychosomatics could only be established as a distinctive discipline with these innovative concepts that defined its epistemological status between somatic medicine and psychiatry.

Modern psychosomatic medicine has evolved as both a critique and an expansion of the biomedical (BM) model, characterized by a reductionistic and objectifying approach to the patient, the preoccupation with the body and disease as primarily biologically conceptualized entities, together with the neglect of the psychosocial and subjective concerns of the patient as a person (2). By contrast, the psychosomatic approach offers a comprehensive, interdisciplinary field providing a clinical, theoretical, and institutional framework for holistic considerations of the patient as a singularly embodied person (3–5). Thus, psychosomatic medicine forms a distinctive discipline and is not a subspecialty of psychiatry (4, 6). However, psychosomatic medicine is multidisciplinary and extends to all areas of medical care (4).

Engel (1), who developed the biopsychosocial (BPS) model, observed that key concepts that are not explicit have an unconscious power over physicians’ thoughts and behaviors. They form part of the fabric of education and are for granted, and reflected in textbooks and institutions. Lipowski (2), Fava et al. (4) and Henningsen (7) emphasized the need to develop the concepts of psychosomatic medicine. Van Oudenhove and Cuypers identified the “risk of semantic and conceptual confusion” regarding the mind–body relationship, criticizing the BPS model’s conceptual underdevelopment and noting “the scientific model (including basic concepts, assumptions and rules) underlying psychosomatic medicine has remained to a large extent implicit” (5).

Despite this, too little attention has been paid to the clinical impact of BPS concepts in education and training and to their systematic analysis as an autonomous field. This paper calls for increased awareness of the clinical significance of concepts in psychosomatic education, training, and research.

After general considerations on the definition of the term concept and on the clinical significance of concepts, I argue in favor of the importance of conceptual competence and research and then address the special nature of psychosomatic concepts through their reference to the mind-body problem and the BPS model. Finally, ways of teaching conceptual competence are discussed.

## 2 The notion of concept

“What disturbs and alarms man, are not the things, but his opinions and fancies about the things.” (8)

Opinions and fancies about the things refer to the underlying concepts of things. The nature of the concept “is one of the notoriously contested topics in the philosophical tradition from antiquity to contemporary debate” (9). Concepts are the “building blocks of thoughts” (10) and might be understood as a linguistic tool that, in some aspects of a thing, inevitably ignores other aspects of the same thing (10). Human percepts always contain concepts (11): Through concepts, we sense a surface as rough or soft, or experience a situation as just or unjust.

Following Descartes, concepts were considered to have a representative function, mirroring reality in the mind (12). The philosophy of the 20th century, however, recognized that linguistic phenomena have designative and performative functions (13–15). Man, a “language animal” (15) uses language not only as a medium of representation but also of action, reaching agreement, or constituting meaning.

**Figure 1:**
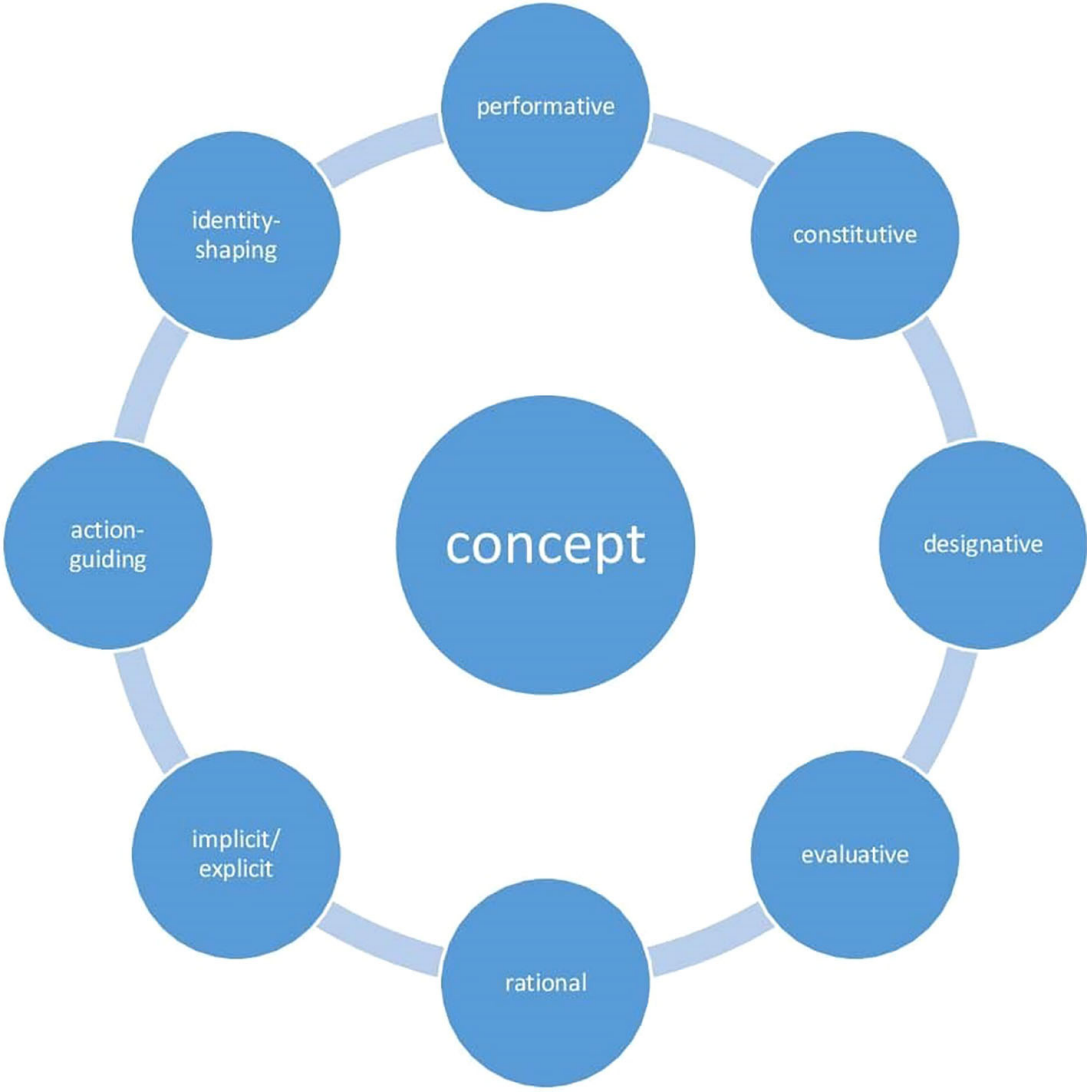
Dimensions of the concept.

Concepts are rational phenomena (16) as well as social phenomena (17). Moreover, they have an evaluative and action-guiding dimension, modeling how humans treat each other and themselves. Concepts are crucial to the formation of personal identity (15). Concepts do not merely describe the world and its objects as detached from us but also constitute the objects that they semantically reflect while shaping our attitudes toward them. As multidimensional entities (**Figure 1**), concepts describe and shape our relationships to ourselves, to others, and to the world we live in.

Concepts are constituents of a theoretical model (2), a conceptual framework (18, 19), or a paradigm (17). A paradigm represents a specific theoretical outlook that is based on a particular epistemology and methodology, forming “the conceptual network through which scientists view the world” (17). What a scientist “sees” depends “both upon what he looks at and also upon what his previous visual-conceptual experience has taught him to see” (17). Clinical concepts are those tools of language by which we try to understand clinical phenomena theoretically.

## 3 The clinical significance of concepts

“There is nothing more practical than a good theory” (20)

### 3.1 Concepts structuring the clinical encounter and care

In medicine, theories, concept formations, methods, and research strategies rest upon the defining objectives of medicine, namely not to harm, to prevent illness, to alleviate suffering; to maintain, promote, or restore health, to prolong human life, and to treat the patient as a person (21, 22). Psychosomatic concepts and methods are used where they can help us best achieve the aims that we strive for. The outcomes of our practice are closely linked to conceptual models and thinking, as concepts are among the most effective *tools* in medicine (24, 25). They enable specific medical practices and shape a particular attitude of care.

Concepts influence the clinical situation but do not fully determine it. The use of concept depends on its explicit and implicit content and on the clinician’s understanding of it. This is shaped by the clinician’s orientation, experience, and attitude and by nonclinical background assumptions (26–28). The use of concepts is always subjective. Dreher (27) noted that there is a triangular relationship between concept, phenomenon, and clinician.

### 3.2 Concepts as constitutive parts of the hidden curriculum

Medical education goes beyond the acquisition of knowledge and skills, and medical practice is more than the application of these: “medical training affects the meaning of medicine” and “occurs on cognitive, affective, and experiential levels” (23).

The notion of medicine’s hidden curriculum, developed in the sociology of medical education in 1960s, shows how the values and objectives of medicine could be undermined by its educational, organizational, and work practices (29, 30). For example, studies have shown that a number of medical students and residents who enter on training with idealism and compassion later show reduced empathy in clinical training due to the distress stemming from the hidden curriculum (31, 32).

Concepts, terms, and language use in medicine are an important part of the fabric of training practices (33, 34). A critical investigation of the hidden curriculum would entail a critical assessment of the hidden aspects of medicine’s fundamental concepts.

## 4 Conceptual competence in the clinic

The clinical significance of concepts suggests that a competent dealing with clinical concepts should be promoted. In the following, this competence will be referred to as conceptual competence. It is an ability that needs development on an interdisciplinary basis. Important preliminary work for psychiatry was done by Aftab et al. (35, 36). They argue that the disregard of conceptual expertise “has led to a state of conceptual impoverishment of mainstream psychiatric practice” (36), although though there is a growing body of interdisciplinary conceptual work on health care issues. Conceptual competence entails “the transformative awareness of how background philosophical assumptions–held by clinicians, patients and members of society at large–shape various aspects of clinical practice, research and education” (36).

Conceptual competence comprises four essential aspects: (1) exploring conceptual assumptions and questions that underlie clinical practice; (2) acquirement of a philosophical vocabulary to explore those conceptual assumptions; (3) engagement in structured philosophical discourse; and (4) fostering conceptual humility, of acknowledgment of the tentative nature of scientific and philosophical findings, and the value of pluralism in explanatory models (35, 36).

Based on Aftab’s notion of conceptual competence, I expand the understanding of conceptual competence, with reference to pragmatism. Pragmatism primarily addresses the practical consequences of human thought, defining ideas and concepts in terms of their effects. The “rational purport of a word or other expression, lies exclusively in its conceivable bearing upon the conduct of life” (37). Our beliefs could thus be called “rules for action,” so that “to develop a thought’s meaning, we need only determine what conduct it is fitted to produce: that conduct is for us its sole significance” (38).

Pragmatism is particularly suitable for developing a practical science such as medicine, where theory is subordinated to practical goals. According to Bernstein (39), the central themes of pragmatism are important in the work of many important philosophers, such as Heidegger and Wittgenstein, due to its fundamental critique of Cartesianism. Bernstein (39) identified several dominant and interrelated themes of pragmatism:

1. *Anti-foundationalism*: pragmatism rejects any absolute foundation for knowledge not in favor of relativism but an understanding of inquiry as “a self-correcting enterprise that has no fixed absolute beginning point or absolute end point” (39). This anti-foundationalism does not deny truth, objectivity, or moral validity.
2. *Fallibilism*: understanding inquiry as a permanent self-correcting enterprise means that all knowledge claims are fallible, corrigible, and questionable.
3. *The community of inquirers*: pragmatists consider inquiry a social practice for which the validity of one’s knowledge claims is assessed by exposing them to others’ criticism. Inquiry as a self-correcting enterprise happens and takes place in a community. Thus, valid beliefs can only be obtained and examined intersubjectively.
4. *Pluralism and contingency*: pragmatic pluralism is an “engaged fallibilist pluralism” (39), because it entails willingness to listen to others with an open mind and without prematurely appropriating what is foreign and translating it into our own vocabularies. This involves transposing “ourselves into understanding persons and ideas that are radically different from ours, and to have the courage and humility to enlarge our horizons in light of new evidence and new encounters with others” (39). Pragmatism is related to a strong sense of contingency as basic to human life.
5. *The agent’s perspective and the continuity of theory and practice*: pragmatists stress the agent’s perspective, distancing themselves from what Dewey called the “spectator theory of knowledge” (39). Inquiry involves searching, critical testing, experimentation, correction, and problem solving.

Against the background of previous considerations and recalling the central themes of pragmatism, conceptual competence can be defined as a transformative awareness of the complexity and multilayered nature of human concepts, which are not only descriptive but also evaluative and action-guiding, recognizing the clinical effectiveness of concepts as tools with explicit and implicit meaning, with an intimate connection between theory and practice.

With this, conceptual competence involves an awareness of the tentative and fallible nature of any knowledge, its social nature, and its plurality and incompleteness. This enables rational agreement with respect to convincing and plausible images together with ideas about the self, the other and the world, along with a common realization of goals.

## 5 The example of psychosomatic medicine

### 5.1 Mind-body relation

Psychosomatics falls epistemologically between somatic medicine and psychiatry. In psychosomatics, more than any other medical discipline, examination of the mind–body distinction is implicitly and explicitly at the center of clinical practice, theory, research and education. The mind–body problem concerns the question of how to relate material things (e.g., organs, tissues, and molecules) with mental things (e.g., consciousness, thoughts, intentions, memories, needs, and emotions). The question of this relationship is linked to questions concerning both ontological status and causal links (40). Over the history of this problem, theoretical forms have evolved to deal with this. The prevailing theories are the monistic and dualistic positions (5, 40, 41).

**Box 1** provides a sketch of an overview.

**Box 1:** Theories of the mind–body relationship.

**1. Monism as materialism and physicalism**: Materialism holds that the human being, including the mind, consists of complex biophysical matter and that human characteristics can be entirely explained by natural laws, as described by the natural sciences; mental states can thus be regarded and observed from outside as purely physically determined. 1.1. **Eliminativism**: This is the most radical position in materialistic monism. It denies the existence of mental properties. 1.2. **Reductive materialism (Psychophysical Identity Theory)**: This approach posits the identity of mental and brain states, holding that the mind can be completely reduced to the physical properties of the brain. Mental states have neither independence nor causal effectiveness on the physical on their own. 1.3. **Non-reductive and supervenience materialism**: For this view, mental properties cannot be fully reduced to physical properties, representing functional states that supervene on biophysical states. Such supervenient mental states have thus a certain independence from physical states, as well as a certain causal effectiveness of its own on the physical field. **2. Dualism:** Although materialism in the form of physicalism is the predominant position in contemporary science, dualism is nevertheless a relevant position. The two subpositions should be distinguished: 2.1. **Substance dualism:** This perspective holds that there exist both nonphysical (mental), immaterial substances that are separate from physical (bodily) substances (Cartesian dualism is the best known example of this—Descartes argued for mutually interwoven interaction between the two substances). 2.2. **Property dualism:** This begins with from the assumption that only physical substances exist and that these possess both physical mental properties. Such properties differ from each other and are not mutually reducible. In property dualism, two positions can again be discerned that differ in terms of the autonomy and causal power attributed to the mental: 2.2.1. **Epiphenomenalism:** This takes the position that the mental is an epiphenomenon of the physical and that it is completely dependent on it, being devoid of any causal force in the physical realm. 2.2.2. **Emergentism:** This contrasting view holds that the human nervous system may generate mental properties that are then basically independent of any underlying physical properties. Emergentism postulates for the mental an autonomous status and its own causal effectiveness on the physical (“downward causation”). **Epiphenomenalism** is thus compatible with the requirement of the causal closure of the physical realm, denying mental causation in that realm, whereas **emergentism** complies with mental causation in the physical domain through denying the causal closure of that domain.

Outline of the different monistic and dualistic concepts are in bold.

Materialist models are generally predominant. The prevailing strategy involves reducing the Cartesian mind–body dualism to its material aspects, locating the mind inside the brain. In modern, scientifically informed world views, the psychophysical unity (not identity) of a person’s mind and body is inarguable, and minds wholly belong to the physical world. However, as Weiner (40) plausibly notes, the mind–body problem is a philosophical question that cannot be resolved scientifically. Current concepts, such as dual aspectivity, 4E-cognition, embodiment, the mind–brain problem, the brain–body problem, and so on (42) are, often contrary to what is claimed, only versions of the mind–body problem: “So the mind–body problem remains with us” (40).

### 5.2 BPS model

“The totality of human life [ … ] cannot be

The object of any scientific research [ … ]

Everything that we can grasp is finite and

Isolated and not the man himself” (43).

The BPS model, as described by Engel (2) in 1977, proposed as a model for all of medicine, currently is probably *the* paradigmatic conceptual model for psychosomatic medicine (2–5, 7). The modern BPS model is a way of dealing with the mind–body problem, not of solving it. Engel created his model as both a critique and an expansion of the traditional BM model, considering the biological, psychological, and social aspects of health and disease in their reciprocal interactions, thus introducing a multifactorial frame of reference (4).

Several criticisms of the BPS model can be distinguished: it is vague and not testable (5, 44–46); too general and eclectic (46, 47); a mere juxtaposition of causal factors without an integrative framework (48, 49); and not methodological and inapplicable in daily practice (22, 45, 50).

Notwithstanding this criticism, scientific evidence on the plausibility of the model is continuously accumulating (4, 51–54), and it is continuously undergoing conceptual development through various initiatives: referring to nonreductive, supervenience physicalism to fill conceptual lacuna in the BPS model (5); taking greater consideration of cultural aspects (7); taking specific systems into account that should be investigated precisely along the various interfaces (53); emphasizing scientific and clinical particulars, not generalities (52); investigating single pathways among biological (B), psychological (P), and social (S) factors more intensively for their relevance for subjective well-being (S→P and B→P) and objective health outcomes (P→B and S→B) (54); taking greater account of the first- and second-person perspectives, particularly with respect to somatic processes, and of the findings of predictive processing and the embodied self (55).

Engel’s model can be interpreted in different ways, which may be seen as a strength or a weakness. It does not and should not cover “the totality of human life” (43). It postulates consideration of biological, social, and psychological aspects, but does not determine the interrelationships among them (7). For none of these aspects can a causal privilege be postulated over the others, and their influence on the etiology, course, and treatment of human illness is not always equally important either (56, 57). The relative weighting of each of these aspects could vary among disorders and patients, as well as across patients who have with a disorder that is terminologically the same as well as throughout the patient’s life history (4, 58). Engel’s model does not define any differences between the mere addition of these aspects and their reciprocal integrations. The terminologically distinct models—BM vs. BPS—=form a continuum both in theory and practice.

### 5.3 Psychosomatic concepts using the example of cardiovascular disease

Clinical concepts become psychosomatic concepts by their use in a psychosomatic context. Psychosomatic concepts are those concepts that enable psychosomatic care, based on an integrative understanding of body and mind that takes into account the biopsychosocial interactions. The content of these concepts is drawn from all areas of medicine. This will be briefly outlined using the example of CVD.

CVDs are a group of disorders of the heart and blood vessels such as coronary heart disease (angina, myocardial infarction, arrhythmia), heart failure, cerebrovascular disease (TIA, stroke), or peripheral arterial disease. They are often associated with a build-up of fatty deposits inside the arteries (atherosclerosis) and an increased risk of blood clots leading to a reduced arterial perfusion. In recent decades, these pathophysiological processes underlying CVD could have been embedded in a psychosomatic perspective on the basis of a large number of empirical good-quality data. Current scientific statements and guidelines from the American Heart Association for CVD (59) and the European Society of Cardiology for chronic coronary syndromes (60) summarize a large number of these findings. Both positive and negative psychosocial factors can be of considerable impact on cardiovascular health:

Positive affect, happiness, optimism, sense of purpose, mindfulness, gratitude, emotional vitality, and psychological well-being are empirically correlated to higher levels of cardiovascular health. In contrast, adverse childhood experiences, stressful life events, a high level of perceived stress in the family and at work, low socioeconomic status, social isolation and a lack of social support have been shown to have a negative impact on cardiovascular health. Anger, hostility and pessimism, as well as depression, anxiety, post-traumatic stress disorder (PTSD), schizophrenia, bipolar disorder, and borderline personality disorder also are associated with a detrimental effect on cardiovascular health (59–62).

Psychosocial factors may affect cardiovascular health through direct biological alterations or indirect effects on behaviors. Negative psychological health, for example, is associated with unfavorable behaviors such as smoking, physical inactivity, poor eating, weight gain, or medication noncompliance. Direct biological alterations that are empirically associated with negative psychosocial factors, are activation of the hypothalamic-pituitary-adrenal axis, dysregulation of the autonomic nervous system, inflammation, hypercoagulability, increased arterial stiffness, or endothelial dysfunction (59, 60).

A psychosomatic view of the disease allows for psychosomatic treatment planning and care (68). Treatment of negative psychosocial factors, depression, PTSD, and anxiety through pharmacotherapy, psychotherapy, collaborative care, stress management, and positive psychology programs can alleviate symptoms and improve quality of life in some patients, and there is increasing evidence for improvement in cardiac outcome (59, 60, 63).

Psychosomatic concepts open up the clinical approach not only to the psychosocial dimension of physical illness, but also to the somatic dimension of mental illness. This is all the more important as mental illnesses are associated with significantly increased somatic morbidity and mortality (64, 65). People with severe mental illnesses (SMI) have a life expectancy that is approximately 15-25 years shorter than the general population. The majority of the excess premature mortality is caused by cardiovascular disease. The cardiovascular mortality rate in people with SMI is more than twice as high as in the general population (61). There is concerning evidence that secondary prevention has been far less successful in the SMI population than in the general population, which has led to a widening of the mortality gap in recent years (66).

## 6 Conceptual competence in research

Conceptual competence is advanced through conceptual research, that can be generally defined as a range of research activities focused on systematic analysis, clarification, and advancement in the meaning and use of concepts (25, 27). Suitable for this are the exploration of the historical context of a concept’s origin, the development of a concept in relation to its paradigm, the current meaning and use of a concept in clinical practice, and a critical reflection of its meaning and use in relation to the objectives to be achieved with the help of the concept. Conceptual competence and research are not mere accessory tools but are instead essential and independent parts of clinical competence and education.

The quality of patient care and empirical research is strongly influenced by the quality of the underlying theoretical concepts. Empirical research *applies* concepts to the empirical pursuit of research objectives, while conceptual research *investigates* concepts themselves (27). Empirical and conceptual research are closely intertwined and mutually stimulating. New empirical findings shed new light on conceptual insights and provide conceptual refinement and precision; more precise concepts enable the discovery of new empirical evidence.

So far, conceptual research is not yet standardized procedure. It is: not a method but by a topic (27). It can be generally defined as a range of research activities focused on the systematic analysis, clarification, and advancement of the meaning and use of concepts (25, 27). Conceptual reflection entails reflection on concepts through concepts.

Conceptual research is both descriptive and normative in its approach (67): descriptively analyzing the concepts that we have and normatively searching for the concepts we need for the care we provide. The descriptive procedure explores how the meaning space and the actual use of a concept might apply to an everyday concrete clinical situation—how a concept shapes clinical practice and its actual impact on treatment. Descriptive conceptual research examines concepts’ explicit meanings, their implicit contents and dynamics, and their appropriateness to the goals in pursuit of which they are used. Normative conceptual research seeks the advancement, refinement, or development of clinical concepts that can support pursuit of the intended goals.

## 7 Conclusions

Psychosomatic medicine has developed into an independent discipline through its development of distinctive concepts that give rise to its identity between somatic medicine and psychiatry. The current identity of psychosomatic medicine has been largely shaped by the BPS model. However, this model requires continuous conceptual efforts to avoid becoming a dogma and to meet the challenges and needs of the rapidly transforming medical, scientific and sociocultural environment. Conceptual research provides a profound reflection and examination of the basic concepts underlying and determining the quality of clinical practice, education, and empirical research to improve the guiding concepts. Conceptual competence is necessary for the appropriate and effective application of concepts in clinical practice.

## Data Availability

The original contributions presented in the study are included in the article/supplementary material. Further inquiries can be directed to the corresponding author.
