# Corrigendum: Conceptual competence in medicine: promoting psychosomatic awareness in clinics, research and education

**DOI:** 10.3389/fpsyt.2025.1575091

**Published:** 2025-03-04

**Authors:** Dirk von Boetticher

**Affiliations:** Department of Psychosomatic Medicine and Psychotherapy, University Medical Centre Göttingen, Gööttingen, Germany

**Keywords:** conceptual competence, conceptual research, mind-body relation, biopsychosocial model, psychosomatic medicine

In the published article, there was an error in the **Funding** statement. The author declared that no support was received for the research, authorship, and/or publication of the article. The corrected **Funding** statement appears below:

“The author(s) declare financial support was received for the research, authorship, and/or publication of this article. The author acknowledges support by the Open Access Publication Funds of the Göttingen University.”

In the published article, there was an error in **5 The example of psychosomatic medicine**, *5.3 Psychosomatic concepts using the example of cardiovascular disease*, Paragraph 5. The first sentence was missing a reference. It previously stated:

“A psychosomatic view of the disease allows for psychosomatic treatment planning and care.”

The corrected sentence and missing reference appear below:

“A psychosomatic view of the disease allows for psychosomatic treatment planning and care (68).”

The author apologizes for these errors and states that this does not change the scientific conclusions of the article in any way. The original article has been updated.

## References

[68] Herrmann-Lingen C Albus C Titscher G eds. Psychocardiology. A practical guide for doctors and psychologists. Berlin: Springer (2022).

